# Societal changes bring about the imperative to readjust the medical gaze

**DOI:** 10.3325/cmj.2023.64.147

**Published:** 2023-06

**Authors:** Hrvoje Barić

**Affiliations:** 1 *Croatian Medical Journal,* Zagreb, Croatia; 2 Department of Neurosurgery, Zagreb University Hospital Center, Zagreb, Croatia

Panta rhei

Heraclitus

Everything flows. The saying was first uttered millennia ago but still holds true, albeit with slight modifications. The rate of flow is accelerating. Also, we keep going in circles and fall into the same difficulties, over and over again. Medicine is not immune to change, far from it. Moreover, the selective pressures are deepening and driving adaptive processes within medicine. In trying to comprehend these processes, one turns to the history of thought. What did our predecessors have to say about the ever-flowing nature of our profession?

## Foucault was right…

The term “medical gaze” was introduced by Michel Foucault in the 1960s. It describes the process by which doctors objectify patients and consider certain (biological) aspects of the individual while ignoring others. The “gaze” is applicable not only to doctors, but to medicine as a whole. It is a useful concept for defining the position of the profession within a wider social context. This concept can help us evaluate the dominance of medicine over certain aspects of human activity, the scope and limits of its authority, the background processes of its legitimation, etc. The ascent of the modern medical profession was, according to Foucault, linked to the establishment of the “gaze,” which was in turn possible due to the prominence of the scientific worldview. Thus, societal change gave birth to the medical profession.

The focus of medicine has shifted dramatically since its modern rebirth during the Enlightenment as an applied scientific discipline: from curing diseases to preventing them, promoting health, augmenting it, and, finally, pursuing longevity in our current age. Given these tectonic shifts, it comes as no surprise that the scope of the problems considered “medical” has evolved comparatively. This has offered many opportunities (for the profession and the patients alike), yet it has also opened many questions. In the face of a rapidly evolving social environment, where does one find firm ground to orient and assess the rising challenges faced by the medical profession, and scientific communication and publishing? Are there any commonalities between the changes in technology, society, and economics, and the landscape of “medical affairs”? Does there exist a common thread between disruptive technology, rising anti-science sentiment, wealth inequality, changes in our global physical environment (1-3) and the goals of medicine? What is the best single approach to face the multitude of obstacles, and prepare for future ones? If societal changes gave birth to medicine, could they also provide remedies for its ailments?

Will societal changes this time force medicine into a rebirth? Rather than bend it, break it so it can be rebuilt anew? If so, new structural foundations will be laid outside medicine and deep within the superstructures of society.

## … so was Štampar

Another 20th century giant spent a lifetime pondering the place of medicine in the world. Andrija Štampar was a Croatian public health visionary, whose ideas have shaped public health policies worldwide (4). Štampar advocated universal, accessible, and just health care. He campaigned for interdisciplinarity and against exclusive dominance of the medical profession in providing health care. His ten principles, published a century ago, hold true to this day, a fact surely testifying to his greatness. Throughout his career, Štampar emphasized the importance of the wider social context of medical care. His last principle is exemplary: “The principal fields of action of a physician are human settlements and not laboratories and consulting rooms.”

What would Štampar make of the world today? Since his time, we have eradicated malaria (one of his many professional efforts) in many parts of the world, yet new species have been introduced into the environment, and a cure to counter them still needs to be discovered. Today, a social media post aimed at spreading misinformation might be as dangerous as a bite of a plasmodium-infected mosquito in 20th century Europe. The vector might be different, yet other features are strikingly similar: both are communicable diseases with a wide reach, quick populational spread, subtle initiation, short incubation period, and crippling damage on a societal level.

In what way is Štampar relevant to all of this? If we are true to his teaching, the way to approach the modern patient is to comprehend his or her “settlement,” including its digital climate, social relations, economic circumstances, and biological strains. To tackle the problem is to step outside the “laboratories and consulting rooms” and appreciate the patient in the context of where and how he or she lives.

## Both are relevant today

Foucault and Štampar, a philosopher and a doctor, both examined the role of the medical profession in a wider context. The former started his analysis from a perspective outside of medicine, the latter from within, yet their lines of thought converged. The medical profession, across its levels, is contingent upon societal structures and their dynamics.

Štampar left a permanent impression on global medical policies and institutions. In his home country, his legacy is still nurtured, foremost at the century-old School of Public Health at the Zagreb University Medical School, which bears his name. The School’s efforts remain focused on developing an encompassing view of the patient in order to meet the challenges of the day. Their striving translates, among other achievements, into academic output aimed at discussing the rising complexities of our profession. The *Croatian Medical Journal* shares their mission (5,6).

Everything flows, medicine included. Maybe the way to keep a sound perspective is to take a step back and look at the bigger picture. Foucault and Štampar would doubtlessly agree.
